# Stakeholders’ Perspectives, Needs, and Barriers to Self-Management for People With Physical Disabilities Experiencing Chronic Conditions: Focus Group Study

**DOI:** 10.2196/43309

**Published:** 2023-12-18

**Authors:** Eric Evans, Ayse Zengul, Amy Knight, Amanda Willig, Andrea Cherrington, Tapan Mehta, Mohanraj Thirumalai

**Affiliations:** 1 Department of Family and Community Medicine Heersink School of Medicine University of Alabama at Birmingham Birmingham, AL United States; 2 Department of Health Services Administration School of Health Professions University of Alabama at Birmingham Birmingham, AL United States

**Keywords:** self-management, physical disabilities, physical disability, chronic condition, chronic illness, mental health, physical activity, barrier, disability, chronic, technology, interview, data, symptom, support, digital, development, need, perspective, barrier, qualitative, focus group, assistive technology, assistive technologies

## Abstract

**Background:**

While self-management programs have had significant improvements for individuals with chronic conditions, less is known about the impact of self-management programs for individuals with physical disabilities who experience chronic conditions, as no holistic self-management programs exist for this population. Similarly, there is limited knowledge of how other stakeholders, such as caregivers, health experts, and researchers, view self-management programs in the context of disability, chronic health conditions, and assistive technologies.

**Objective:**

This study aimed to obtain insight into how stakeholders perceive self-management relating to physical disability, chronic conditions, and assistive technologies.

**Methods:**

Nine focus groups were conducted by 2 trained facilitators using semistructured interview guides. Each guide contained questions relating to stakeholders’ experiences, challenges with self-management programs, and perceptions of assistive technologies. Focus groups were audio recorded and transcribed. Thematic analysis was conducted on the focus group data.

**Results:**

A total of 47 individuals participated in the focus groups. By using a constructivist grounded approach and inductive data collection, three main themes emerged from the focus groups: (1) perspectives, (2) needs, and (3) barriers of stakeholders. Stakeholders emphasized the importance of physical activity, mental health, symptom management, medication management, participant centeredness, and chronic disease and disability education. Participants viewed technology as a beneficial aide to their daily self-management and expressed their desire to have peer-to-peer support in web-based self-management programs. Additional views of technology included the ability to access individualized, educational content and connect with other individuals who experience similar health conditions or struggle with caregiving duties.

**Conclusions:**

The findings suggest that the development of any web-based self-management program should include mental health education and resources in addition to physical activity content and symptom management and be cost-effective. Beyond the inclusion of educational resources, stakeholders desired customization or patient centeredness in the program to meet the overall needs of individuals with physical disabilities and caregivers. The development of web-based self-management programs should be holistic in meeting the needs of all stakeholders.

**Trial Registration:**

ClinicalTrials.gov NCT05481593; https://clinicaltrials.gov/study/NCT05481593

## Introduction

Approximately 25% of Americans have a disability, which is nearly 61 million individuals. About 13.7% of the population experiences a mobility or physical disability, which affects daily activities such as walking or climbing stairs [1]. This demographic is more physically inactive than individuals without physical disabilities [2,3]. The increase in physical disabilities has led to higher rates of chronic conditions compared to individuals without physical disabilities, including obesity, heart disease, and diabetes [4]. For example, individuals with physical disabilities have a significantly higher heart disease rate (12%) compared to individuals without physical disabilities (4%) [1].

Self-management programs are used to manage health conditions. A primary goal of self-management programs is to include the person as a central role in their health and wellness. Within this central role, a person with a chronic health condition is intimately and actively involved with health care providers and the health care system, regularly obtains skills and knowledge to manage symptoms, and increases self-efficacy to handle health-related issues [5]. Additionally, these programs promote self-care, regular physical activity, healthy nutrition behaviors, and medication adherence [5,6]. Within self-management, there are core skills that include problem-solving, decision-making, resource use, health care provider partnership, and action taking [7,8]. These core skills include involvement in all areas of self-management, ranging from identifying symptoms, using web-based resources to learn about safe exercise, and communicating with health care providers [7]. The multifaceted approach of self-management programs has shown significant improvements in chronic conditions, including stroke, diabetes, and hypertension [9-11].

While self-management can be viewed solely from the perspective of the individual, self-management is a collective process that includes a strong social and professional network, including caregivers and health care providers. Self-management is not an individual endeavor experienced by those with physical disabilities and chronic health conditions. In many instances, caregivers are intimately involved in providing daily assistance through various duties, including but not limited to activities of daily living, nutritional needs, medication management, and coordination of care with health care providers [12,13]. Because self-management is complex due to its multiple components, caregivers can experience numerous barriers that prevent adequate caregiving, such as being overworked, economic status, and the lack of educational resources [12]. Therefore, an understanding of the self-management from the perspective of caregivers is warranted. Additionally, self-management is not confined to the home setting but also includes health care professionals and experts who initiate, guide, inform, and direct medical care. Individuals with chronic health conditions typically maintain regular contact with health care professionals or seek out relevant resources to ensure that symptoms and disease progression are well managed. Health care professionals and experts typically emphasize skills and behaviors that are typically associated with self-management, including obtaining regular physical activity, drinking water, following a healthy diet, and symptom tracking [14]. However, because individuals with chronic health conditions may not be able to maintain regular contact with health care professionals or experts, it is necessary to understand the perspectives of experts on self-management programs.

Despite improvements in self-management programs, less is known about how these programs affect individuals with physical disabilities [10,15]. Unfortunately, there is no such holistic chronic disease management program for individuals with physical disabilities. Compared to individuals without physical disabilities, those with physical disabilities are prone to poorer health outcomes and are more likely to participate in unhealthy behaviors, such as smoking [16]. Although self-management programs are successful, they are fragmented with content targeting specific behaviors or symptoms rather than using a holistic approach. For example, programs may focus on a specific health-related domain (ie, medication adherence) but may not focus on other significant health components, including nutrition, physical activity, or other health behaviors.

Since self-management is a collaborative endeavor between individuals, caregivers, and health care providers, understanding collective viewpoints is needed to ensure that future programs include acceptable, comprehensive content for all users [17]. Therefore, the purpose of this study was to gain insight using focus groups into how stakeholders perceive self-management in the context of physical disability, chronic conditions, and assistive technologies. The stakeholders included individuals with physical disabilities who experience chronic conditions, caregivers, health experts, and researchers.

## Methods

### Ethical Considerations

This study was approved by the institutional review board of the University of Alabama at Birmingham (IRB 300009485). Participants provided consent to participate following a screening questionnaire. All study data, including identifying information, audio recordings, and transcriptions, were deidentified. Upon the completion of each focus group, participants were compensated with a US $25 gift card.

### Participants

Upon approval from the university institutional review board, all participants were purposively recruited using advertisements through the National Center on Health Physical Activity and Disability website and its social media channels. Based on how individuals answered screening questions, they were filtered into 1 of the 8 groups. This study was conducted in the United States. Interested participants completed screening and consented to participate in a focus group (Table 1). Individuals with diabetes, heart conditions, and lung conditions were specifically chosen due to the high prevalence of these conditions [18-20]. Focus groups were prescheduled, and only participants who were able to attend on the dates were eligible. Participants were required to be at least 18 years old and attend a Zoom (Zoom Technologies, Inc) call with up to 20 participants per group.

**Table 1:** Stakeholders of focus groups (n=47).

Group	Stakeholders	Value, n (%)
1	People with disabilities and diabetes	6 (13)
2	People with disabilities and heart conditions	5 (11)
3	People with disabilities and lung conditions	7 (15)
4	Caregivers of people with disabilities and diabetes	8 (17)
5	Caregivers of people with disabilities and heart conditions	2 (4)
6	Caregivers of people with disabilities and lung conditions	1 (2)
7	People with disabilities and any chronic condition (eg, no specific health condition required)	6 (13)
8	Health experts and researchers	12 (26)

### Focus Group Procedures

Under the oversight of the principal investigator (MT), postdoctoral fellow (EE), and predoctoral student (AZ), 2 trained facilitators conducted the focus groups. No prior relationship between participants and facilitators was established. MT, EE, and AZ were present during the focus groups but did not actively participate to avoid bias. The facilitators were given a study description and met with the research team prior to the focus groups to discuss study goals and interview guides. Three semistructured interview guides were developed for each stakeholder group and contained questions regarding chronic conditions and physical disabilities, self-management, psychological perspectives, and assistive technologies (Multimedia Appendix 1). Focus groups lasted up to 90 minutes, and participants were encouraged to speak freely. Field notes were collected during each focus group. No additional focus groups were conducted as data saturation was achieved. Focus groups were conducted between August and September 2021.

### Qualitative Analysis: Data Collection and Analysis

In terms of ontology, we adopted the constructivist grounded theory (CGT) approach within an epistemological position of subjectivism. We acknowledge that researchers cannot be fully objective and there is an interrelationship between participants and researchers. By adopting this approach, we focused on developing the themes through inductive analysis of the raw data gathered from our participants [21].

Using this approach, 3 main themes—perceptions, needs, and barriers—were identified. Perceptions included thoughts and attitudes toward self-management and self-management–related behaviors. Needs included elements that are required to assist in overall care and barriers included elements of health that were difficult to maintain. Using a constructivist grounded approach and inductive data collection allowed us to uncover many nuanced understandings by providing comprehensive and contextually embedded explorations of the stakeholders’ experiences [22]. Focus groups were audio recorded and transcribed using a web-based transcription service. Transcribed files were imported and coded by MT, EE, and AZ using the Nodes feature in NVivo software (NVivo 12, QSR International). A coding tree was developed using the identified themes, and the transcripts were reviewed by the research team and were matched with the field notes. Elements of the coding tree are represented by the categories in Multimedia Appendices 2 and 3 (sample coding tree from NVivo provided in Multimedia Appendix 4). Any coding changes were determined in discussions among MT, EE, and AZ. All disputes were resolved by MT. All findings were presented to the project advisory board, a panel of 12 individuals with chronic conditions and physical disabilities, caregivers, health experts, and researchers.

## Results

### Overview

A total of 268 participants were eligible to participate in the focus group study, with 47 individuals participating due to scheduling availability. Three groups were part of the study: individuals with physical disabilities and chronic conditions (n=24), caregivers of individuals with physical disabilities and chronic conditions (n=11; Table 2), and health experts and researchers (n=12). Additional relevant quotes are provided in Multimedia Appendix 2.

**Table 2:** Descriptive characteristics of caregiversa in focus groups.

Characteristics		Caregivers (n=11), n (%)
**Mobility disabilities and health conditions in which caregivers provide care**		
	Spinal cord injury	1 (9)
	Muscular dystrophy	1 (9)
	Diabetes	6 (55)
	Asthma	2 (18)
	Food allergies	1 (9)
	Attention-deficit/hyperactivity disorder	1 (9)
	Down syndrome	1 (9)
	Asperger	1 (9)
	Congestive heart failure	1 (9)
	Parkinson disease	2 (18)
	Depression	1 (9)
	Posttraumatic stress disorder	1 (9)
	Chronic pain	1 (9)
	Chronic obstructive pulmonary disorder	1 (9)
	Renal failure	2 (18)
**Role of caregivers**		
	Informal (spouse, family member, etc)	10 (91)
	Professional	1 (9)
**Areas of caregiving provided**		
	Medication adherence	6 (55)
	Dietary assistance	5 (45)
	Medical appointments	7 (64)
	Activities of daily living (driving, bathing, dressing, etc)	5 (45)
	Emotional support	2 (18)

^a^Unrelated to participants with physical disabilities and chronic health conditions.

Participants with physical disabilities indicated that they experienced other chronic health conditions outside of diabetes, heart conditions, and lung conditions (Multimedia Appendix 5). All caregivers were informal (ie, spouse and family member) and were unrelated to any participants in any other focus group. Health experts included health and wellness experts (n=2), researchers (n=6; administration, investigator, etc), and community outreach and development experts (n=4).

### Perceptions of Stakeholders

#### Overview

Stakeholders were asked multiple questions to obtain thoughts about self-management and self-management programs. By using the semistructured interview guides, participants shared their experiences, either performing self-management or providing self-management services. By using CGT and inductive analysis, perceptions of stakeholders became a resulting theme from the focus groups. Relevant codes and explanations of the codes are provided in Multimedia Appendix 2.

#### Individuals With Physical Disabilities and Chronic Conditions

Many participants expressed that physical activity was an important component of their usual self-management. Walking or wheeling was the most mentioned activity, followed by water-based activities such as water aerobics. Participants expressed a desire to join a community center where they could participate in group classes or use exercise equipment. Nutritional behaviors were also discussed as an important element of self-management. Emphasis was placed on eating fruits and vegetables, reducing high-carbohydrate foods, taking vitamins and supplements, avoiding excess sodium, preparing meals, and reducing sugar intake.

(I) try to move around as much as I can, because I think moving is good for me. I think I also just try to do as much as I can, while accepting that some days are going to be better than others, in terms of how much I can do.

Interestingly, individuals indicated that managing mental health was also a significant component of self-management. Anxiety and fear were the most common emotions cited. In addressing anxiety and fear, familial support and religion were the primary ways to manage emotions. Other forms of managing mental health include meditation, yoga, breathing exercises, and regular social interactions.

When I think of health, I think of mental health, but then my physical health, too, and kind of how it all goes together. I'm trying to figure out why I react certain ways to everything I have going on lately with my emotions and mental wellbeing.

Participants also shared what motivated them to manage their health. The most common motivation was the desire to live a long life with family and friends. Similarly, social support received from friends was an additional motivation for maintaining health. Additionally, working with health care providers was provided as a mode of self-management.

I see my primary care once a month. I see my therapist once a week, my cardiologist every three months. I get an EKG every, every month when I see my primary care. I see my COP doctor every three months, three to five months.

Technology use was discussed as a mechanism for self-management. Participants stated that using mobile apps helped with physical activity, nutritional habits, and communication with health care providers. Web-based support groups were perceived to be helpful for communicating with others with similar experiences. Additionally, participants used teleconferencing technology for group exercise classes. When asked whether web-based self-management programs are helpful, most indicated that such programs would be beneficial for daily self-care. Specifically, peer support and social capabilities were highlighted as the most useful components of web-based programming. Additionally, a program that would be regularly updated with the latest health recommendations would be considered for daily use.

I use my different technologies a lot, like my phone. I have an iPhone, a iPad, and so I use my like reminders features. I use the different apps, like the CVS app to manage my medication, you know, check and see how many refills I have.

#### Caregivers

Caregivers expressed views about self-management programs and caregiving duties. Most shared that having an educational resource for daily caregiving would be beneficial. Specifically, caregivers believed that self-management programs with educational resources about medications would aid in managing health, such as medication reminders, side effects, and dosages.

It’s mostly arranging for medications and appointments and taking him to appointments. (It) can be a full-time job. But I’m lucky I have help. My family’s very supportive.

When asked for perspectives on web-based self-management programs, caregivers believed technologies and programs would be able to aid in daily responsibilities. Specifically, they preferred web-based platforms that include peer support and educational resources for a wide variety of health conditions.

I would love something like that especially if we can interact with people like this and talk. I like to talk and learn about other people and you know what they're going through. I have a lot of empathy and that's just the person that I've always been. I feel like I can learn from someone else. Even if it's not the same exact disease, I can talk to somebody who has fatigue, and they'll understand, and maybe they can give me ideas.

#### Health Experts and Researchers

Health experts provided perspectives on their role in self-management. For example, experts shared how they provided assistance to individuals, including teleconferencing technology for at-home exercise and modifiable exercise equipment during exercise sessions. Additionally, due to the COVID-19 pandemic, experts emphasized telehealth for interacting with their clients.

So, what I will say is I was shocked when, when lockdown happened originally and I had to transfer everything online, how quickly all of my clients were just like, “Yep. I do Zoom now.” Like, I've really not had, a ton of barriers in terms of user ability with Zoom. Of course, internet access is an issue for anyone. But generally speaking, I think, like all of my clients did a great job with that.

For working with caregivers, health experts noted that maintaining independence may be a barrier as the individual may not seek assistance during an exercise or therapy session. Caregivers explained that the individual they care for may not want assistance during a session. Therefore, the ability to work with the caregiver could be hindered. Educating staff about family dynamics may be helpful for caregiving [23].

Health experts were asked to give ways they help clients who have chronic conditions and physical disabilities. The most common form of assistance given included sharing contact information. Through communication, experts could answer health-related questions concerning exercise or nutrition.

Ongoing motivation and support. So, I'm readily available by email. I also, really love connecting people. So, just like she said, if we can't physically be in the same room, are you comfortable, if you guys share your information? Can I have you guys swap emails?

Researchers provided perspectives on self-management programs. Due to the COVID-19 pandemic, researchers perceived that delivering programs in a hybrid or completely digital version was beneficial. Researchers noted that individuals who could not attend in-person programs due to inaccessible transportation would be able to participate remotely. However, broadening access to programs requires internet capabilities, which may be a barrier. Additionally, researchers noted that web-based programs need to accommodate visual and hearing impairments.

We’ve got some trainings that we flipped to virtual because of COVID, and they’re staying virtual because people are able to have greater participation and connect in a better way, and that has made people kind of stay in programs longer, so you might get better retention.

#### Summary of Perceptions

In summary, stakeholders emphasized the importance of healthy behaviors including physical activity and healthy eating. Interestingly, stakeholders included managing mental health in the context of self-management as an important component for overall health and relied on social support. Many also provided positive statements related to using technological aides to assist in their daily self-management programs. Elements within technological aides cited included educational resources and web-based social support groups.

### Needs of Stakeholders

#### Overview

Stakeholders were asked questions related to areas of self-management and self-management programs that lacked essential resources or support to provide individuals with the optimal level of self-management, whether it applied to personal or professional experience. By using CGT and inductive analysis, the needs of stakeholders became the second theme in the focus group study.

#### Individuals With Physical Disabilities and Chronic Conditions

Participants shared areas of self-management that they wanted to improve. The primary focus was symptom management. Specifically, participants wanted to learn how to identify triggers that preceded symptoms such as pain or fatigue. Increasing physical activity was another area where participants wanted to improve. Most wanted to increase time spent walking or wheeling, while some preferred structured exercise. Similarly, identifying triggers for symptom management and avoiding foods that exacerbate symptoms were common.

For me, the two main things that I wish I was better at is tracking how I’m feeling so I can try and identify triggers or things that make my pain level, for example, better or worse.

Participants provided several suggestions on needed elements for self-management programs. Peer-to-peer support was the most cited feature that participants wanted. Participants indicated that being able to communicate with other individuals would facilitate their ability to manage their symptoms. Additionally, a regularly updated program with educational resources was discussed as being relevant for a web-based self-management program.

I think that an online health application would be wonderful. I would love to be able to see any features as developed as the app or the program itself went along. Peer-to-peer support, online support with possibly the specialists. That could be an optional thing… application reminders such as: medication reminders, pharmacy pick up reminders, coordinating with your doctor or specialist should any problems arise.

#### Caregivers

Caregivers discussed needed areas of self-management to provide adequate care. Understanding symptoms and medication was the primary need. They desired a centralized mechanism to retrieve relevant information on various conditions and physical disabilities. Additionally, caregivers conveyed their desire to have emotional well-being resources.

There are things that I, we come across that are just brand new to us and, some of the things that my immediate family and immediate support system haven’t come across at all. So just having more knowledge and more access to resources that have that knowledge is something that I strongly desire.

Caregivers provided suggestions on what components of web-based self-management programs would assist them in their roles as caregivers. The most common element included a resource bank with educational information about conditions and disabilities. Additionally, caregivers recommended peer-to-peer groups, where caregivers could interact with other caregivers who experience similar challenges.

#### Health Experts and Researchers

Health experts discussed areas of self-management where they assist clients. The most common area was technology use. Specifically, health experts helped clients use teleconferencing technology and features such as closed captions or specialized audio settings. For in-person assistance, health experts would help their clients navigate physical and environmental structures. Subsequently, experts would have conversations about client preferences and goals.

I would say that the people with disabilities or the chronic conditions, they not only need the support that a physical medication or something like that, but emotional and moral of support is also important to them. So basically, what to do is a support group and one of the people that we engage there, we, try to encourage each other. Also, the medication that we make sure that everyone is getting the right medication.

Researchers were asked to share their thoughts on needed elements of self-management programs. Person centeredness was the most common component recommended. Allowing individuals to customize for their needs would be essential for overall well-being. The researchers also expressed the need for regularly maintained programs with evidence-based recommendations. Lastly, implementing user-friendly language was suggested to make programs more appealing.

It’s really important that people with disabilities be involved in all aspects of these things, planning leadership roles. There is such a big difference having people with disabilities thinking about the program and then developing the program, and doing the outreach, and leading the programs.

#### Summary of Needs

Individuals with physical disabilities and chronic health conditions indicated that for them to improve in self-management behaviors, accessible and educational resources would be beneficial. Similar thoughts were expressed by caregivers, in addition to understanding health conditions, symptoms, and medication side effects and interactions. Health experts and researchers emphasized that resources provided to individuals with physical disabilities and chronic health conditions and caregivers should be individualized and participant centered.

### Barriers of Stakeholders

#### Overview

The stakeholders were asked to provide their perspectives on areas within self-management and self-management programs that prevent the ability to successfully manage health behaviors and health conditions. Through using CGT and inductive analysis, the third theme elicited from the data was barriers of stakeholders. Relevant codes and explanations of the codes are provided in Multimedia Appendix 2.

#### Individuals With Physical Disabilities and Chronic Conditions

Participants discussed different barriers that affect daily self-management. Symptom management was common among nearly all participants. Most participants indicated that symptom severity makes it difficult to perform daily activities, such as housekeeping and cooking. The most common symptoms were fatigue and pain, followed by mental health issues, including depression, fear, and anxiety. Most anxieties and fears stem from losing independence and a shortened lifespan. The economic burden of self-management was another barrier to maintaining health. Participants indicated that covering costs for medication, even with insurance, is a financial barrier to health. Additionally, participants expressed that healthy food options are more expensive than processed foods and that they have difficulty determining what foods are appropriate for their diet.

I would say one of the biggest issues for me that no one has mentioned is financial. I live at the poverty level. It definitely freaks me out. I mean, I'd be homeless if I didn't have help. So that's like one of the main concerns I have. I mean, all my money goes to my secondary insurance and my medications, and I didn't get to work long enough to really end up making that much money.

#### Caregivers

Caregivers were asked to share barriers they experienced when providing daily assistance to whom they provided care. Many stated that medical care was the most challenging aspect of self-management, including medications and working with health care providers. The most challenging aspect of medication management was handling multiple prescriptions that were taken at different times throughout the day. Subsequently, prescriptions are regularly updated, which would cause confusion. Similarly, due to having multiple conditions or physical disabilities, managing medical appointments with physicians is a significant challenge. Specifically, understanding the symptoms and side effects of medications is a constant challenge for caregiving. Caregivers also shared fears and anxieties related to self-management. The primary concern was maintaining the balance between providing adequate assistance and allowing maximal autonomy.

My painstaking needle in the foot is medications. It starts picking up the medications, refilling the medications. Then I go home and I have a book this big with about twenty different medications and I have to pull apart every little thing that's on the tab.

#### Researchers and Health Experts

Researchers and health experts were asked to give thoughts on barriers for self-management programs. Stakeholders suggested that health care providers need specialized training to work with individuals with physical disabilities. Specifically, emphasis should be placed on learning to communicate with an individual with a physical disability. Additionally, researchers highlighted the importance of addressing mental health concerns for these populations while not solely focusing on the physical symptoms.

So some people have mental health as a priority because they're coping with the changes to their life given the diagnosis, some people are very focused on pain management, others are focused on, what biomarkers are they getting measured for at the doctor's offices? …but I think making it patient-centered so that people can actually have a say in what they want out of treatment or a program in this case, as opposed to kind of it being dictated to them.

#### Summary of Barriers

In summary, individuals with physical disabilities and chronic health conditions indicated that the symptoms experienced were the most common concerns that prevented adequate self-management, followed by economic concerns. Caregivers experienced challenges in medication management. The health experts and researchers suggested that health care providers lack training to work with those with chronic health conditions, especially those with physical disabilities.

## Discussion

### Principal Findings

The purpose of this study was to gain insight into how stakeholders perceive self-management of health in the context of physical disability, chronic conditions, and assistive technologies. Regular physical activity or exercise was noted as the most important facilitator of good health but also the most challenging activity. Participants indicated that these activities were conducted in individual or group settings. According to the Centers for Disease Control and Prevention, individuals with physical disabilities are less likely to achieve physical activity recommendations, and this effect is increased if one has a physical disability and chronic condition [1,24]. For needs, patient centeredness, continuing education, and emotional needs were deemed important. Including stakeholders in the creation of web-based self-management programming was also considered a necessary element in self-management [25-27]. For barriers, participants with chronic conditions and physical disabilities indicated that managing symptoms was the most prevalent barrier to participating in physical activity such as fatigue and pain. These symptoms have been shown to negatively impact physical activity participation [28,29]. Self-management programs are designed to address different barriers to promote participation in safe physical activity [7]. Research has shown that self-management programs that include physical activity promotion are feasible and effective for individuals with physical disabilities or chronic conditions [30-34]. Thus, any self-management program should include accessible and inclusive physical activity recommendations.

Interestingly, mental health was also perceived to be a barrier to health. Participants shared that they experience emotional barriers, including depression, fear, and anxiety. These perspectives are consistent with research showing that similar psychological elements are common in different disability-related populations, such as multiple sclerosis, spinal cord injury, or stroke [35-37]. The remaining stakeholders expressed similar concerns about mental health. Specifically, caregivers expressed their desire to have mental health resources available to assist family members or friends with emotions stemming from experiencing physical disabilities or chronic conditions. This supports the need to implement mental health education in self-management programs [38].

Caregivers addressed the need for educational content regarding physical activity recommendations, symptoms, and medications. Health experts and researchers also expressed the need for inclusive education. Within the literature, caregivers and experts believe that there needs to be ongoing education that is accessible and inclusive [39,40]. In addition, caregivers expressed physical and emotional stress during daily caregiving. Mental burdens were prevalent as managing multiple conditions causes confusion as they evolve. It is suggested that low levels of caregiving assistance and working outside the home are associated with more health and wellness problems for caregivers [41]. Thus, providing mental health support to both people with physical disabilities and their caregivers can influence the efficacy of self-management programs.

Additionally, caregivers expressed challenges they experienced when managing medication. Understanding medication, side effects, daily administration, and autonomy in using medication were the most common challenges. Caregivers discussed difficulties in understanding how medications worked, especially with other medications. These challenges made managing medications difficult because several medications are needed to manage multiple conditions and disabilities. These findings are consistent with caregivers who handle medications on a daily basis [42]. Because caregivers contribute significantly to the preparation of daily medications, they are likely to experience stress with such duties. Caregivers cited that preparing daily medication and the lack of knowledge add to the burdens they experience. Research has shown that multiple conditions, medications, medication interactions and the lack of medical knowledge make caregiving responsibilities problematic [43-45]. Accordingly, self-management programs should include education about multiple conditions and medications.

Other barriers that were common among participants were economic strains. Caregivers and individuals with chronic conditions and physical disabilities indicated the complexities of navigating the health care system, including Medicare and Medicaid. Additionally, medication costs made sustaining a healthy lifestyle more difficult. These concerns are similar across multiple conditions and physical disabilities [46-49]. Self-management programs should consider serving as a resource in navigating the tough financial landscape.

Stakeholders discussed perspectives, needs, and barriers when incorporating technology into self-management programs. Many individuals with physical disabilities and chronic health conditions indicated that they used mobile apps to assist with monitoring exercise and physical activity, nutrition, and medication adherence. Overall, stakeholders emphasized the benefit of using digital technologies in self-management programs. More specifically, stakeholders expressed support for technology platforms that included peer-to-peer support functions, which would allow users to be able to communicate with other individuals. Individuals with chronic conditions and physical disabilities conveyed that they used teleconferencing technology to attend group exercise classes. Videoconferencing technology is a feasible way to promote physical activity participation [50]. Caregivers emphasized the benefit of using digital technology with educational resources to aid in daily responsibilities. These perceptions are consistent with prior literature indicating support for technology-integrated programs for daily self-management [51,52]. These features have been implemented in web-based programs to promote behavioral change, goal setting, and symptom management [32,53-55]. Several participants noted that although several technologies were available, there was a burden with finding technologies, and many fragmented pieces of technologies were needed to accomplish their goals. Thus, self-management programs should consider providing holistic technological solutions or serving as a resource for finding such solutions.

### Limitations

While stakeholders provided valuable perspectives on self-management, there are limitations that need to be addressed. First, the sample size of stakeholders may be perceived as a limitation; more specifically, the caregivers and the health experts and researchers groups. Next, participants’ responses to questions may have been influenced by a prior participant’s answer [56]. For participants with chronic health conditions and physical disabilities, inclusion criteria for chronic health conditions included diabetes, heart conditions, and lung conditions. The focus group responses may not be fully representative of other health conditions.

### Conclusions

This study sought to gain information from stakeholders who have experience with chronic conditions and physical disabilities. Stakeholders shared common views regarding the importance of physical activity, nutrition, and medication adherence. Interestingly, stakeholders emphasized that mental health was generally missing from self-management programs. Due to the COVID-19 pandemic, stakeholders suggested that web-based self-management programs could be a viable method to manage health. Based on the findings, web-based self-management programming with mental health resources would be beneficial for long-term care. Overall, programs should take a holistic view of the needs of people with physical disabilities and chronic conditions.

## Appendix

Multimedia Appendix 1 Semistructured interview guides for stakeholder groups.

Multimedia Appendix 2 Additional demonstrative quotes.

Multimedia Appendix 3 Code tree for focus groups.

Multimedia Appendix 4 Sample NVivo screenshot for code tree.

Multimedia Appendix 5 Physical disabilities and health conditions experienced by individuals with physical disabilities and chronic health conditions.

## Abbreviations

CGT: constructivist grounded theory

## Data Availability

The data sets generated and analyzed during this study are available from the corresponding author on reasonable request.
