# Supplementary material for: Stakeholders’ Perspectives, Needs, and Barriers to Self-Management for People With Physical Disabilities Experiencing Chronic Conditions: Focus Group Study
Source: JMIR Rehabil Assist Technol. 2023 Dec 18;10:e43309. doi: 10.2196/43309 (PMC10758937; doi:10.2196/43309)
Supplement: Multimedia Appendix 1 [file rehab_v10i1e43309_app1.docx]

| Appendix A. Semi-structured interview guides for stakeholder groups |
| --- |
| Individuals with chronic conditions and disabilities |
| 1. What disability and chronic conditions do you have? For how long? 2. Can you describe what it is like to live with these conditions? 3. How do you currently manage your conditions? 4. What do you find most difficult in managing or coping with your specific condition and disability? 5. How do you overcome those challenges? 6. Can you describe what your current management routine is like for your chronic conditions and disabilities? If yes, how or where did you learn about this routine? 7. What motivates you to continue with your management routine? And what do you hope to achieve with your self-management routine? 8. Are there times when you are satisfied with your self-management routine for your condition? If so, can you tell me about them? 9. Do you have any fears or anxieties about your future? If so, how do you cope with these thoughts and feelings? 10. Are there any areas about your self-management routine that are confusing or hard to understand? If so, can you share what they are? 11. Are there any areas of self-management that you know about, but are not currently doing? If so, what are they? 12. Would an online self-management program help you with managing your chronic conditions or disability? If yes, how so? |
| Caregivers |
| 1. What disability and chronic condition(s) does the person you caregive for have? How long have they had them (condition and/or disability)? 2. In general, what ways do you assist the person you caregive for in managing their condition or disability (daily routines, eating, etc)? 3. Can you describe what a typical day is like as a caregiver? (i.e., like what your daily schedule or responsibilities entail) 4. Are there any areas of caregiving that you find difficult to accomplish when assisting the person you caregive (managing daily activities, emotions, etc.) 5. How do you overcome those challenges? 6. Are there times when you are satisfied with your caregiving routine for the person you caregive for? If so, can you tell me about them? 7. Do you have any fears or anxiety about your future for the person you caregive? If so, how do you cope with these thoughts and feelings? 8. Are there any areas about caregiving that are confusing or hard to understand? If so, can you share what they are? 9. Are there any areas of caregiving that you wish you knew more about? If so, what are they? 10. Would an online self-management program help you in your duties as a caregiver for someone with a chronic conditions or disability? If yes, how so? |
| Health Experts & Researchers |
| 1. What kind of work have you conducted with people with physical disabilities who have chronic conditions? If so, what kind of physical disabilities and chronic conditions? 2. Broadly speaking, what kind of considerations do you make when working with people with physical disabilities and chronic conditions? 3. How do you incorporate preferences or considerations of a person with a physical disability when providing any form of care?” 4. What kind of information would you like to know about the person you are working with before providing your expertise or service? 5. Besides physical activity and nutrition recommendations, are there any other unique elements that you provide for those you are working with?(social, emotional, etc.) 6. How do you provide ongoing motivation and support for those you are working with? 7. How often do you communicate with the people you work with, whether it’s your initiating or the individual initiating? What are the ways you communicate with the people you work with? 8. Do you provide any specific educational content for people with chronic conditions and physical disabilities? If so, where you do retrieve this information? 9. Have you worked with caregivers of individuals with disabilities? If so, what do you believe is your role in working with the caregivers? 10. What is the role of assistive technologies in providing care to the people you are providing care? 11. In your opinion, what are the most important elements of a chronic disease management program? 12. Based on your experience, what are the most important educational areas that should be emphasized more for people with chronic disease and/or physical disabilities? 13. In your opinion, are there any missing elements in chronic disease management programs? 14. Generally speaking, do you think that chronic disease management programs are successful? Why or why not? 15. In your opinion, would an online self-management program be beneficial for those managing their chronic conditions and/or physical disabilities? Why or why not? |
