# Supplementary material for: Stakeholders’ Perspectives, Needs, and Barriers to Self-Management for People With Physical Disabilities Experiencing Chronic Conditions: Focus Group Study
Source: JMIR Rehabil Assist Technol. 2023 Dec 18;10:e43309. doi: 10.2196/43309 (PMC10758937; doi:10.2196/43309)
Supplement: Multimedia Appendix 2 [file rehab_v10i1e43309_app2.docx]

|  | | Appendix B. Additional Demonstrative Quotes | |
| --- | --- | --- | --- |
| Theme | Explanation of Theme | | Demonstrative Quotes |
| Perceptions of stakeholders |  | |  |
| Individuals with chronic conditions and disabilities |  | |  |
| Physical activity participation | Participants’ thoughts on physical activity or exercise, whether they participate in any activity, and their perceptions on its benefits | | “Walking is terrific for me emotionally, physically, mentally”  “But usually over the years, uh, being a manual wheelchair user, um, I've always been able to exercise. Get a lot of exercise just pushing myself. Um, I would just do like light weights and stuff to stay in shape, but I've really had to cut back on that due to the arm overuse injuries.”  “I try to go out for walks when the weather is decent” |
| Medication management | Participants’ thoughts and experiences on managing their medications on a daily basis | | “Um, so for me, um, I try to stay on top of my medications, um, ensuring that, like, you know, I take them on time, and stay on top of refills, uh, because sometimes, you know, I may need to request a refill, you know, a script be sent over to the pharmacy, and then that could take a day or two. And then sometimes I go without my pain meds for a day or two, and that's not good, so I try to stay on top of that kind of thing.”  “I do use a lot of apps. I don’t put a lot of things in. I got one of those smart watches.” |
| Managing mental health | Participants’ thoughts and experiences with managing mental health | | “So just different things I’ve been going through mentally. But overall, I’ve just been trying to use like meditation and yoga and focus on my breathing to help with the anxiety because I’ve had a lot lately.”  “So that’s why I was trying to really focus on like, holistic avenues, like the eating and yoga and stretching and… And it’s actually really helped, and I kind of been mentally adapting into a holistic lifestyle as much as I can.”  “You know, my motivation is to live a long time, be here for my family and friends, and also, there’s a lot of things I want to do in my life.”  “My motivation was just always to be independent in all aspects of life.”  “Uh, the frustrating thing is- is being in the wheelchair not being able to drive or go places where I want to go because I can't afford those m- handicap uh, thing- vehicles. So it's always depending on somebody. And sometimes I get sick and tired of um, using uh, the uh, the- the Medicaid transportation. So, that's my daily frustration sometime. That's about it. I just want to be able to jump in a car, go wherever I want to go, take my grandkids and just go. That's my frustration. Other than that, everything else is great.”  “Um, I find it confusing about mental health. It's very stressful for me. I try to understand it and I just can't. So I ha- I have those difficulties with that.”  “So I've learned just by experience that if I have a fear, I need to face it head on and tackle it. That seems to be the best way. Um, that, and acting on what I need to do to keep myself as healthy as possible, that for me is the best way of tackling the situation to keep my fears from increasing and becoming uncontrollable and obsessive. Because I just don't have time for that.”  “I'd like to be in therapy. I need therapy. I think that will give me a better understanding as what my medical conditions mean to me.”  “I'm having a problem actually finding a therapist and also trying to get like outside help. I don't know if it's just because of the Coronavirus or just because there's lack of, um, professionals. I think there's a lot of like lack of healthcare professionals.” |
| Caregivers |  | |  |
| Assistance offered | Participants’ experiences on what kinds of assistance they provide to those whom they caregive | | “It's mostly arranging for, um, medications and appointments and taking, uh, him to, um, appointments (laughs) can be a full-time job. But, um, I'm lucky I have help. My family's very supportive.”  “Okay. For me, a typical day is, uh, getting up early, uh, preparing her breakfast, uh, checking out her doctor's appointments 'cause we have to schedule that in on our calendar book, um, seeing what doctors she needs to go to, um... just all day long, the schedule of feeding all day long. Cooking and feeding and... you know, seeing about her- seeing if she used the bathroom and all that kind of stuff, just in a row.”  “Um, I prepare breakfast, do the household routines, medications, uh, I let him get up and we do a little walk. Then we both take a nap, we have lunch, um, after lunch I try to make sure he goes outside, sits for a little while. And in between all that we're doing bathroom duty, you know, changing clothes, washing clothes. Everything you can think of, daily living, assisted living, but we're doing it at home.” |
| Satisfaction with caregiving | Caregivers’ perspectives on their ability to successfully assist those whom they caregive on a regular basis | | “I think anytime that I see, um, progress in my son, i- it's a big win. Um, not only for him, but for me as well because it helps me, um, it reinforces that, you know, the techniques and the strategies that we're doing to help him and helping, um, so that's when I really, really feel good about, um, the care that I'm giving them when he's able to come back and say, "Hey, Dad, I, um, was able to do this by myself," or this was scary to me at first, but now I know I can just walk away from it instead of, you know, um, screaming at the top of my lungs. It's, it's what makes me feel good.”  “Um, I think there's great satisfaction when there is time to do things that weren't planned or we're doing something that isn't needed. So I, the immediate thing that comes to my mind is when we're able to just sit on the porch, um, and step away from, you know, this highly regimented schedule, um, just... You know. Or there's a unexpected visitor, um, or we're able to just grab a meal. So anytime we're able to kinda step away from the routine and do something that reminds us of maybe past times, um, is rewarding.” |
| Online self-management programming | Participants’ perception of online technology and resources in their ability to assist in caregiving duties | | “I think it would be beneficial to get, um, the support. You know, it could be ongoing and, you know, [inaudible 01:05:48] the group of peers that's going through similar, you know, situations. And, um, you know, we get other resources and stuff that would benefit the ongoing challenges we have.”  “I'd be able like, uh, to be having like, a schedule on, uh, to do the activities. And, uh, uh, also I- I'll say the other thing like, uh, I- it will be really helpful to me like, uh, having a networking of the caregivers who are like, uh, patients who are suffering the same disease like I'm caregiving to. So I- I feel I could be able to share our experiences. I'll be able to share like, uh, different ways on how I've been able to manage or rather like, handle the situations I'll be in, too. So I- I feel it's good to have networking and I've been trying a lot to even search on social media if I could really get any group which is like, uh, it belongs to the caregivers.”  “At least it will be good to share ideas with people who are like, doing the same thing. So I, I feel it will be good with that. So, on the issue of like, uh, getting the notification. I feel it's really good because I'd be able to get notification, maybe it shows all other times which I should administer the medication to my relative. And also, notification on reminders like, uh, the day or the, the time you have to attend to the hospital or rather, visit the doctor. So it's really good and, uh, it be able not to forget each ... any time.” |
| Health Experts & Researchers |  | |  |
| Working with caregivers | Participants’ perspectives on the importance of collaborating and working with healthcare providers to successfully manage chronic health conditions | | “I think that my interaction with care partners is very much dependent on the family dynamic and also on the diagnosis. So, for example, um, I could work with two people with CP and one could be like, "Mom, please leave," right? Like I've had that happen where it's like, the client that I am working with actually doesn't want mom to be part of the session. And so, I'm, I'm trying to mitigate that. Versus, another person who like really enjoys having mom or dad there and, and they want to participate. And their everyone, sometimes someone else comes in and everyone's doing the class, (laughs), especially if it's like over Zoom, right?”  “Yeah. Um, I do work with staff mostly. Um, one is sometimes their communication. Um, what I mean by that is, uh, I'm gonna not t- you know, try to be okay about this. It could be someone from a different culture and sometimes you can't, they don't understand what you're trying to say and how things need to be done for that individual. So it's easier, I mean, it's easier if you can, to, to include them, so they can, you know, see. Sometimes we've done, um, videos as well, so that way they can bring it back to the staff and train the staff. 'Cause what we find is that, the individual needs to have consistency on how it is done so there are no injuries. That is such, um, a big thing. Um, and that they're transferred in a way, depending on where they need to go to do the exercise and things like that.” |
| Assistive technologies | Participants’ perspectives on using assistive technologies in regular self-management | | “So that's, it's just like, um, that's why this work is so incredible and I'm so grateful to be part of this group. Um, in terms of assistive technology, the way I use things in classes are different. So, for example, um, for some, for, they can't necessarily ... They don't have grip strength, so I'll sub out a hand weight for a cuff weight, which is a different kind of assistive technology. If folks use Therabands, again, if they don't have grip, we have special Therabands that have cuffs, so people can wear it like a wristlet. Um, tying your Theraband to something, as opposed to using it free standing, that's another way that you can manipulate the assistive technology wheelchairs. Um, you, you had mentioned that.” |
| Telehealth usage | Participants’ perspectives on using telehealth technology to provide healthcare services for individuals with physical disabilities who experience chronic health conditions | | “So I've treated on telehealth for physical therapy, but also I've taught group fitness and private fitness. So I kind of have different, like a few different perspectives. I think there's a lot of accessibility that we probably all have thought about, which is like, for us too, right? Like I can join in. You guys are in Alabama, I'm in Philadelphia, right? I couldn't have done this focus group unless you guys were holding it on Zoom. And who knows if you would have two years ago? Maybe you would. Um, so there's that. So I've been able to, to have clients that are across the country.” |
| Thoughts on online self-management programs | Participants’ perspectives on using online self-management programs for managing health conditions and symptoms | | “I think this could have real potential. Of course, uh, um, a hybrid model would offer benefits, but I have also seen the reach and the engagement that can be, uh, facilitated and also, sometimes, better accessibility for participants.”  “In terms of having it online, um, is really helpful for people with chronic illnesses that have fatigue as a major side effects, um, for vasculitis, either from the disease itself or from a lot of the medications. Uh, fatigue is huge, and so it's a lot of like, you know, thinking you're gonna make it somewhere and not being able to. So having, you know, something that's self-paced and something that you can do at home really lets you circumvent that. So I think that that would be a really good tool.” |
| Needs of stakeholders |  | |  |
| Individuals with chronic conditions and disabilities |  | |  |
| Areas of self-management to improve | Participants’ views on personal areas where self-management needs to improve | | “I wish I was better about eating things and avoiding things that make my different conditions worse. For example, like technically my body has trouble absorbing fructose, which is found in so many foods because of high fructose corn syrup. And even like just fruit in general like apples. So like I shouldn’t eat apple sauce, and I really have cut that out of my diet which is sad, because I used to like that a lot.”  “I would like to know more about meal planning. Um, if they were to build something, I wish that they would build something with meal planning and understanding, you know the- the breakdown of carbs and proteins. Uh, that's- that's what I, um, have the hardest time understanding.”  “I wanna get back to eventually walking in a walker. Right now I can't 'cause I'm so weak physically that I just want to be able to get back to, you know, doing more things with my friends, but I can't because my wheelchair is a manual chair, but it's a power assist. So it's really hard for them to put it in their vehicles to go and do stuff.”  “I definitely would like to do more physical activity and workout more.” |
| Suggestions for online-self-management programs | Participants’ suggestions for what is needed for online self-management programs to be deemed successful | | “All of those things together would be absolutely amazing. Um, to be able to put it into a program, um, it would make a person's life, who has medically complex history to deal with, uh, and many issues, it would make their lives more streamlined, less difficult, less time-consuming... Um, you might even be able to relax occasionally, um, if you've got many issues going on in your life as pertains to me. Um, I think that would be fantastic.”  “But I see all these people, like, talking about being mental health coaches, and I always thought that was a bunch of, um, crap (laughs). But now I'm, now that I'm kinda in a weird time in my life, now I can understand where mental health coaches come into play. They're your cheerleader. And I sometimes struggle with the motivation, so having, like, a cheerleader in your corner besides... Somebody kinda outside, you know, your general circle of people. Um, I think that, something like that, the motivator, you know, that motivates more personal, or, something to keep that going t- so that you stay on track.”  “I think people have a hard time with motivation. Um, I know I struggle with that all the time. Um, some type of way to keep people motivated to try to stay on track, um, with what they need. Whether it's, um, like a mentor, like, like, like you're partnering up with somebody through the app to kind of cheer each other on? Um, that might make some good motivation. Kind of like a peer mentor, but, you know, whether they're the mentor, you're the mentor, you're just partnered up kind of with, with somebody randomly just for that. Um, you know, a cheerleader in your corner. Um, something like that.”  “If- if I was to create something, I would create a- a, um, comprehensive center. That comprehensive center would be something that people would come there and find support, such as possibly a virtual doctor. They would be there to find, um, help for medicines. They would be able to look up side-effects of medicines. They would also be able to reach out for referrals. They would also be able to, um, find support groups. They would be able to reach out and find a psychologist. They would also be able to reach out and find help for other diseases, such as diabetes, um, ketoacidosis, strokes, heart attacks, um, res- high blood pressure. They would also be able to, uh, reach out and find dietary plans. They would also reach out and be able to find, um, you know eye clinics. They would able to reach out and find, um, all these support that they needed, such as exercises.” |
| Caregivers |  | |  |
| Areas to increase caregiving knowledge | Participants’ need to increase in knowledge about health conditions experienced by those whom they caregive | | “Okay, I- I would say it will also be really good for me to get like, uh, more information about the disease she is ailing from. And, uh, I will say it is good for me, like, uh, to know how to manage it. Yeah, getting that information and, uh, also the correct information, because a- at times we find like, uh, I'm getting too much information and, uh, considering I'm a layman in medical terms, and also like, uh, the health, health subjects or rather, health subjects. So if I like, I don't understand so much on how to manage the disease at time. So it will be really difficult for me to know how I would be able to really help her at times of need, so I just have to keep in touch with the doctors each and every time, trying to verify, trying to visit different website, trying to dig more information on how I could contain this disease.”  “For me it's really along the lines of having the base that I did already as a mental health provider but not having the base of information and knowledge that I needed to to maneuver the system for what he needed. It actually took me having all but a breakdown in front of his psychologist to say, "Look, this are all the things we've done. What am I not doing so that I can be able to better maneuver the caregiver component of what he needs?"  “I can't narrow it down to a single area. I would say I would want know more across the board. Um, and I wish that there was a clearer path to resources instead of, um, I just feel like so much of what I've discovered is only because of a coworker or a family member who's been through something similar, or a church member. So it's a lot of word of mouth as opposed to, I don't know, case management or, or some, some mechanism to navigate, um, resources more efficiently.” |
| Suggestions for online self-management programs | Caregivers’ suggestions for resources specifically designed to help caregivers provide optimal, regular assistance | | “I just think you, uh... also, you just need somebody to vent to. If somebody would just listen for a minute, it- it doesn't take all day, just a little bit, just to vent to that person. If they were knowledgeable in whatever the ailments, these- that the people have, if... And then if they could just vent- let you vent and then you share something with them, they could maybe tell you something back and that would help you. I don't know.”  “Absolutely, it will. Like, I'd be able like, uh, to be having like, a schedule on, uh, to do the activities. And, uh, uh, also I- I'll say the other thing like, uh, I- it will be really helpful to me like, uh, having a networking of the caregivers who are like, uh, patients who are suffering the same disease like I'm caregiving to. So I- I feel I could be able to share our experiences. I'll be able to share like, uh, different ways on how I've been able to manage or rather like, handle the situations I'll be in, too. So I- I feel it's good to have networking and I've been trying a lot to even search on social media if I could really get any group which is like, uh, it belongs to the caregivers.” |
| Health Experts & Researchers |  | |  |
| Special considerations for working with individuals with disabilities | Experts and researchers’ thoughts for necessary elements to consider when working with individuals with physical disabilities | | “So the other important point is about plain language, and how information gets presented in pictorial form, written form, and alternate formats. That’s a very important point for both children and adults that we should note.”  “Very specific tailored language, depending on what the person is going through.”  “I guess, for some of the times for adaptions, some of my individuals also need staff with them. Because sometimes they can't always move their limbs in the right way, to get the stretch or the things that they need. Um, so that can either be in a pool setting or at home, it's range of motion type stuff. Um, the other thing my individual use are, that could be a stander, I'm assuming many of you know what a stander is. Um, but they can use that or a gate trainer, 'cause sometimes they can't stand up on their own. So they have, it-it's similar to a walker, but it holds you in a lot better so you can walk on your own. Um, so we use adaptions like that.”  “So basically I would say that, you see, the people with disabilities or the chronic conditions, they don't not only need the, uh, maybe support that a physical medication or something like that, but you see, emotional and moral of support is also important to them. So basically, what to do is a support group and, uh, and one of the people that we, um, engage there, uh, we offer these, uh, we, uh, try to encourage each other. Also the medication that we make sure that everyone is getting the right medication.” |
| Relevant elements in self-management programs | Participants’ thoughts on needed elements for self-management programs for individuals with physical disabilities who experience chronic health conditions | | “So some people have mental health as a priority because they're coping with the changes to their life given the diagnosis, some people are very focused on pain management, others are focused on, you know, what biomarkers are they getting measured for at the doctor's offices, and can they get those down? So I think, you know, obviously, it's in conjunction with your p- medical provider, but I think, uh, making it patient-centered so that people can actually have a say in what they want out of treatment or a program in this case, um, as opposed to kind of it being dictated to them, I think, is very important.”  “It's really important that people with disabilities be involved in all aspects of these things, planning leadership roles, um, uh, uh, the do- I... you know, there is such a big difference between having people with disabilities, uh, and, you know, and thinking about the program and then developing the program, and doing the outreach, and leading the programs.”  “We have had, um, a lot of success when it is not just, um, whole person or all diseases, but I do think, um, involving the family in any kind of chronic disease self-management, um, is really, um, helpful in maintaining success.” |
| Missing elements in self-management programs | Participants’ views on elements that are not present in current self-management programs that would benefit the target population | | “I'd also add that there has to be a way to update these programs pretty regularly, um, especially for some of the rare, uh, chronic illnesses or disabilities. You know, you have everything kinda set the way that it was where the information is the way that it was, and it doesn't take into account anything that's been done recently.”  “So the other important point is about plain language, and how information gets presented in pictorial form, written form, um, and alternate formats. That- that's a very important point for both children and adults that we should note.” |
|  |  | |  |
| Barriers of stakeholders |  | |  |
| Individuals with chronic conditions and disabilities |  | |  |
| Barriers and challenges to staying healthy | Participants’ thoughts on personal barriers that prevent the ability to adequately manage their chronic health conditions | | “And the cost of it. The cost of it is a struggle. To try to eat healthy, it's very, very expensive.”  “Um, I would say one of the biggest issues for me that no one has mentioned, um, is, uh, financial. I mean, I live at the poverty level. Um, it definitely, it totally, it freaks me out. I mean, I, I'd be homeless if I didn't have, um, help. Um, so I mean that- that's like the main, one of the main concerns I have. I mean, all my money goes to my secondary insurance and my medications and I just didn't, I didn't get to work long enough to really end up making that much money. “  “I'm having a problem actually finding a therapist and also trying to get like outside help. I don't know if it's just because of the Coronavirus or just because there's lack of, um, professionals. I think there's a lot of like lack of healthcare professionals.” |
| Nutritional challenges | Participants’ barriers and challenges in regularly eating healthy | | “Also like myself, I- I worry, like I'm not eating the right things for my diabetes. Like I used to go to a nutritionist and now, um, they're hard to find. They had, um, they had one in my grocery store that kind of worked with patients, and then they had one at my local hospital, but they're so inundated with people that they really can't see you very often. So I have that, I try to read up on it, but it's really hard to like make sure you're eating the right things.”  “I wish I was better about eating things and avoiding things that make my different conditions worse. Uh, for example like technically my body has trouble absorbing um, fructose, which is found in so many like um, foods because of high fructose corn syrup. Um, and even like just fruit in general like apples. So like I shouldn't eat apple sauce, and I really- I have cut that out of my diet which is sad, because I used to like that a lot. But like limiting how many coffees I have in a day.” |
| Caregivers |  | |  |
| Challenges in caregiving | Caregivers’ barriers in providing high quality caregiving | | “So everything is changed, and trying to get them to understand, and work together, and they're basically in the house more often together, so there is more conflicts, um, than it's typically should be, you know, because everybody was going on other activities and now they kinda just stuck together most of the time. So I would say this is the most challenges, challenging part at this time.”  “Um, for me, it's more of the, uh, eh, emotional support side. Um, it's something that's a given, but m- my son's condition, he can get freaked out, um, by things that my other two kids... I have three children. Uh, my other two kids will see as completely normal. But, um, it could be something very simple, very normal for them and us, um, but to him it's just, um, terrifying. So that's been one of the most challenging parts of, um, his care, be- just providing that emotional support when, when he really needs it.” |
| Confusing areas of caregiving | Areas of caregiving that caregivers perceive to be confusing or challenging that make their duties difficult | | “So my answer would just be like the sheer amount of paperwork, um, and trying to navigate, navigate MediCare, Medicaid, power of attorney, all of that. Um, and I just always find myself thinking there's some real issues with access and equity. Um, because, you know, I may be able to do certain things or have the time, or the resources to do those things with my mother, but someone else may not have computer access, or have transportation or have, um, the means to just navigate of all the... kind of the red tape that goes with dealing with multiple physicians, and therapies, and medication, and all of that”  “There are a few things that are confusing. In my experience, it's less with medical staff and more with other individuals and getting them to understand, you know, um, what my son's situation is and, um, explain to others around him, even his siblings, you know, why, um, he interacts with things differently sometimes. I know that may not be the exact answer you're looking for, but that's been one of the most, uh, um, challenging parts in my experience.” |
| Health Experts & Researchers |  | |  |
| Motivational strategies | Strategies used by health experts that motivate individuals to continue regular self-management behaviors | | “Ongoing motivation and support. Um, so, I'm readily available by email. Uh, I also, I really love connecting people. So, if we can't physically be in the same room, are you comfortable, if, if you guys share your information? Can I have you guys swap emails? And then I've had folks who like, um, didn't know a support group existed, and they joined because they knew someone else. At the end of the day, my role as a teacher, and this is true of anything, whether it's movement or not, is to empower that person to take it with them. Like they don't need me, at the end of the day.” |
| Challenges and obstacles | Participants’ perspectives on global challenges and obstacles that prevent individuals with physical disabilities and chronic health conditions to perform self-management behaviors | | “I wear two hats, right? Because I, too, am a person with a disability. And so capacity of organizations to serve people with disabilities is a problem. And we're not talking from just the point of view of physical access and getting inside the building, there's a gap in staff training, we've failed in a few respects about classroom inclusive culture, so training staff adequately to bring in people with a variety of disabilities to engage effectively in program offerings, uh, would be one area that I would identify.” |
