# Supplementary material for: Stakeholders’ Perspectives, Needs, and Barriers to Self-Management for People With Physical Disabilities Experiencing Chronic Conditions: Focus Group Study
Source: JMIR Rehabil Assist Technol. 2023 Dec 18;10:e43309. doi: 10.2196/43309 (PMC10758937; doi:10.2196/43309)
Supplement: Multimedia Appendix 3 [file rehab_v10i1e43309_app3.docx]

| Code Tree for Individuals with Physical Disabilities and Chronic Health Conditions |  |
| --- | --- |
|  | Barriers to staying healthy  Being active  Challenges to staying healthy  Contemplating to be health  Effective medication management  Healthcare provider  Health-related anxieties  I want to be better at…  Managing mental health  Medical device  Motivation  Not being active nutritional challenges  Nutritional strategy  Overcoming barriers  Satisfaction  Strategy to staying healthy  Symptom management  Symptoms experienced  Technology usage |
| Code Tree for Caregivers |  |
|  | Areas to increase caregiving knowledge  Assistance offered  Online self-management (helpful)  Online self-management (suggestions)  Challenges in caregiving  Confusing areas of caregiving  Fears and anxieties and coping strategies  Overcoming challenges  Satisfaction with caregiving |
| Code Tree for Health Experts and Researchers |  |
|  | Addressing personal and environmental concerns  Assistive technologies  Client populations  Communication strategies  Information needed about client  Motivation strategies  Provided educational content  Special considerations  Telehealth usage  Topics of interest  Working with caregivers  Challenges or obstacles  Educational emphasis  Experience  Important elements in chronic disease management  Missing elements in chronic disease management  Success of chronic disease management programs  Thoughts on online self-management programs |
