# Supplementary figures and images for: Stakeholders’ Perspectives, Needs, and Barriers to Self-Management for People With Physical Disabilities Experiencing Chronic Conditions: Focus Group Study
Source: JMIR Rehabil Assist Technol. 2023 Dec 18;10:e43309. doi: 10.2196/43309 (PMC10758937; doi:10.2196/43309)

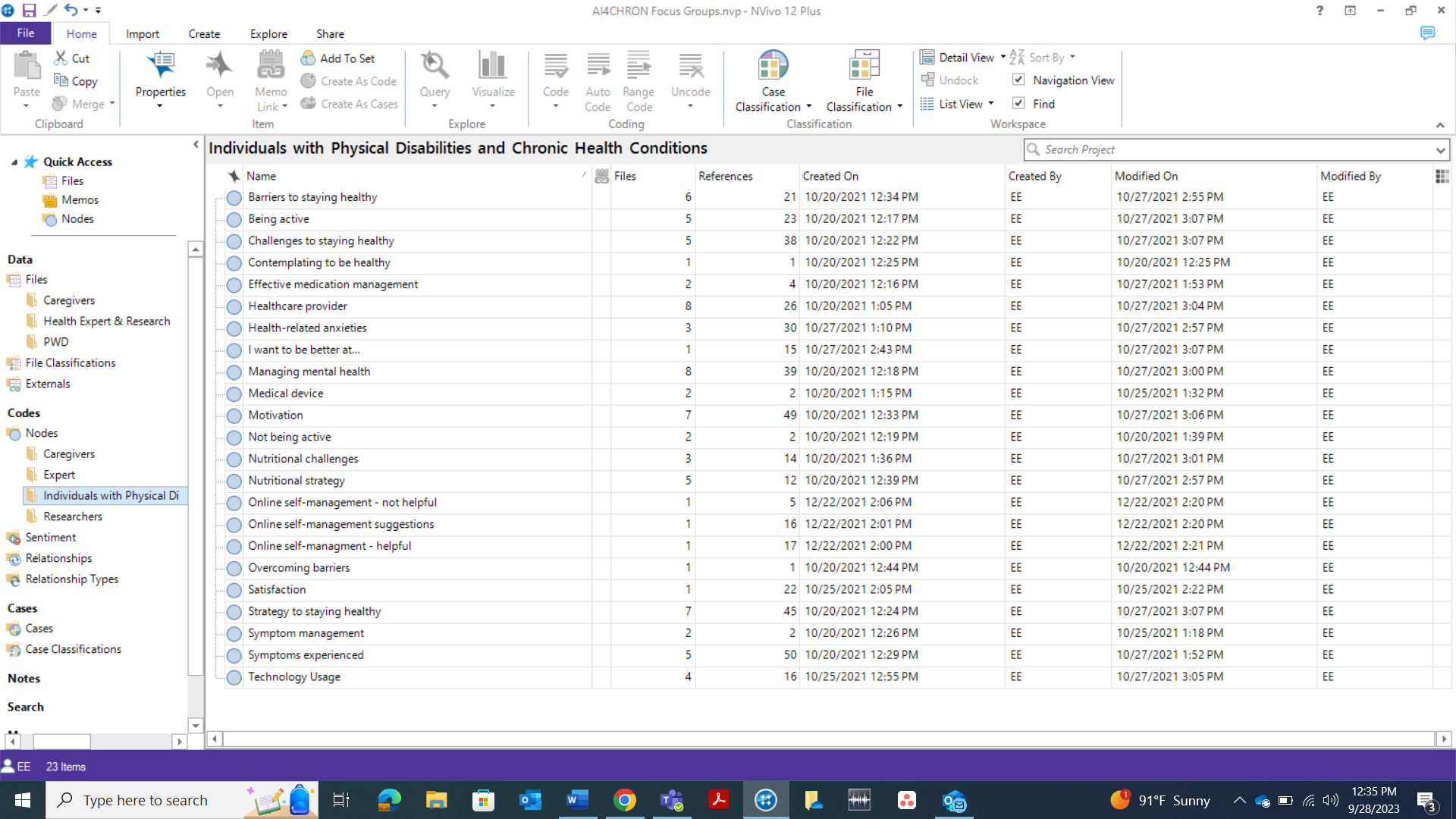

Supplement: Multimedia Appendix 4 [file rehab_v10i1e43309_app4.docx]
