# Supplementary material for: Stakeholders’ Perspectives, Needs, and Barriers to Self-Management for People With Physical Disabilities Experiencing Chronic Conditions: Focus Group Study
Source: JMIR Rehabil Assist Technol. 2023 Dec 18;10:e43309. doi: 10.2196/43309 (PMC10758937; doi:10.2196/43309)
Supplement: Multimedia Appendix 5 [file rehab_v10i1e43309_app5.docx]

| Category | Condition | Number of participants with chronic condition |
| --- | --- | --- |
| Mobility/Physical Disabilities/Musculoskeletal |  |  |
|  | Fibromyalgia | 1 |
|  | Mixed connective tissue disease | 1 |
|  | Scoliosis | 1 |
| Psychiatric |  |  |
|  | Cognitive damage | 1 |
|  | Depression or anxiety | 7 |
|  | Mental health condition | 2 |
|  | Post-traumatic stress disorder | 2 |
| Cardiovascular |  |  |
|  | Bradycardia | 1 |
|  | Cardiovascular disease | 3 |
|  | Hypertension | 2 |
|  | Stroke | 2 |
| Pulmonary |  |  |
|  | Pulmonary fibrosis | 1 |
|  | Pulmonary hypertension | 3 |
|  | Asthma | 5 |
|  | Chronic obstructive pulmonary disease | 4 |
|  | Restricted lung disease | 1 |
| Neurological |  |  |
|  | Epilepsy | 3 |
|  | Narcolepsy | 1 |
|  | Moebius syndrome | 1 |
|  | Spinal cord injury | 1 |
|  | Multiple sclerosis | 1 |
|  | Chronic pain | 2 |
| Degenerative |  |  |
|  | Mixed connective tissue disease | 1 |
|  | Rheumatoid arthritis or arthritis | 2 |
|  | Degenerative disc disease | 1 |
|  | Nerve damage | 1 |
|  | Cerebral palsy | 2 |
| Metabolic Disorders |  |  |
|  | Diabetes | 6 |
| Immune System Disorders |  |  |
|  | Immunocompromised | 1 |
|  | Acquired Immunodeficiency Syndrome | 1 |
| Visual Conditions |  |  |
|  | Blindness or visual loss | 2 |
| ^a^Participants experienced multiple conditions | | |
